# National Insurance: The Distribution of the Medical Benefit Fund

**Published:** 1913-10-18

**Authors:** John Tod Thyne

**Affiliations:** 101 Earlsfield Road, Wandsworth Common, S.W.


### National Insurance: The Distribution of the Medical Benefit Fund.

Sir,—Your leading article in your issue of October 4 has been much appreciated by the non-panel members of the medical profession. On page 18, however, of the same number, referring to the £1,000,000, being the money which has accumulated through insured persons not having selected a doctor, you say: "This sum, in our opinion, undoubtedly belongs to the doctors as part of their remuneration, although they have not actually rendered any service to the particular insured persons involved."

Now, Sir, it was because the *British Medical Journal* had the hardihood to express similar views in its issue of May 31, 1913, that we—the Wandsworth members—resigned our membership of the Association. We look upon this suggestion as simple robbery—first, of the insured persons; and, secondly, of the non-panel doctors. A large number of the insured deeply resent not having been allowed free choice of doctor. They decline to go to panel doctors, and go to their own doctors and pay them privately to attend them. They are never going to any panel doctor, and to suggest that their money—8s. 6d. per annum—is to be given in a present to doctors who have broken their pledges and gone on the panels is simply monstrous, and is strongly objected to on the part of the non-panel men, who have stood to their guns, in spite of heavy loss of income. Mr. Danckwerts, K.C., has ruled that "the practitioners on the panel are entitled to be paid at so much per head of the insured persons on their list, and that the London Committee could not lawfully pay more, and were not entitled to make presents or distribute largess among the panel doctors."

Now, Sir, we loyal non-panel doctors feel that we have been robbed by the Government and by the panel men who have broken their pledges, and we also feel that the British Medical Association has been aiding and abetting these gentlemen in their course of action.

The incomes of the panel men are, I dare say, more than doubled in many instances, but there is public dissatisfaction at the back of all this business, and the people are only waiting patiently for the General Election to come, when, like the wounded gladiator, they will cry "Arise, ye Goths and glut your ire." . . . .—I am, yours faithfully,

John Tod Thyne.

101 Earlsfield Road, Wandsworth Common, S.W.
October 10, 1913.

[The reference to the £1,000,000 mentioned on p. 18 is made by our Insurance Correspondent, and, as pointed out, no distribution of this money is probable or practically possible until the matter has received the authoritative decision of the Courts, failing the resolution of the Insurance Commissioners to undertake the responsibility themselves, and to decide how and in what manner this money shall be dealt with.—Ed. The Hospital.]

